# The Role of Interactional Quality in Learning from Touch Screens during Infancy: Context Matters

**DOI:** 10.3389/fpsyg.2016.01264

**Published:** 2016-08-30

**Authors:** Elizabeth Zack, Rachel Barr

**Affiliations:** ^1^Department of Psychology, Georgetown UniversityWashington, DC, USA; ^2^Institute for Learning and Brain Sciences, University of WashingtonSeattle, WA, USA

**Keywords:** transfer of learning, touch screens, interactional quality, maternal scaffolding, teaching tool, infant, elaborative parenting style, emotional responsiveness

## Abstract

Interactional quality has been shown to enhance learning during book reading and play, but has not been examined during touch screen use. Learning to apply knowledge from a touch screen is complex for infants because it involves transfer of learning between a two-dimensional (2D) screen and three-dimensional (3D) object in the physical world. This study uses a touch screen procedure to examine interactional quality measured via maternal structuring, diversity of maternal language, and dyadic emotional responsiveness and infant outcomes during a transfer of learning task. Fifty 15-month-old infants and their mothers participated in this semi-naturalistic teaching task. Mothers were given a 3D object, and a static image of the object presented on a touch screen. Mothers had 5 min to teach their infant that a button on the real toy works in the same way as a virtual button on the touch screen (or vice versa). Overall, 64% of infants learned how to make the button work, transferring learning from the touch screen to the 3D object or vice versa. Infants were just as successful in the 3D to 2D transfer direction as they were in the 2D to 3D transfer direction. A cluster analysis based on emotional responsiveness, the proportion of diverse maternal verbal input, and amount of maternal structuring resulted in two levels of interactional quality: high quality and moderate quality. A logistic regression revealed the level of interactional quality predicted infant transfer. Infants were 19 times more likely to succeed and transfer learning between the touch screen and real object if they were in a high interactional quality dyad, even after controlling for infant activity levels. The present findings suggest that interactional quality between mother and infant plays an important role in making touch screens effective teaching tools for infants’ learning.

## Introduction

The launch of the iPad in April 2010 was followed by a rapid and unregulated release of more than 80,000 tablet applications or “apps” tagged as educational in the App Store (Apple, 2016). These inexpensive and accessible programs can easily be downloaded onto touch screen enabled phones and tablets. As such, use of touch screens during early childhood is increasing at a rapid pace (Radesky et al., 2015).

The American Academy of Pediatrics (2013) recommends that parents co-use educational media with their children in limited quantities. Co-using media together allows parents to bridge the gaps in their child’s knowledge of the media content and use of the media device. Parents have not consistently adopted these recommendations and these policies have not yet fully considered use of newer tablet touch screen-based technologies (Neumann, 2015). Parents report co-using more often with their children while watching television compared to using smartphones or tablets (Rideout, 2013; Connell et al., 2015). Parents, teachers, and app developers need more evidence-based information about how to best support children’s learning from touch screen devices (Lerner and Barr, 2014; Hirsh-Pasek et al., 2015; Barr and Linebarger, 2016; Troseth et al., 2016).

There is a small but growing body of literature on learning from tablets and touch screens during early childhood (see Barr, 2013; Troseth et al., 2016). On the one hand, the inherent interactivity of touch screens may facilitate learning, such that learning may be less dependent on parental support. For example, toddlers who have contingent interactions with touch screens transfer learning in an object retrieval task (Choi and Kirkorian, 2016) and learn more words than children who view a non-interactive video (Kirkorian et al., 2016). On the other hand, children may appear to be proficient in their interactions with the device, but this may not allow for them to transfer information beyond the app (Moser et al., 2015; Neumann and Neumann, 2015). Interactive media contexts are increasingly becoming part of the day-to-day environments of infants and their caregivers. It is important to understand whether, and in what ways parent–child interactions may enrich these experiences. We do know a considerable amount about the context of learning with real objects.

Social interaction with parents and other significant adults help to shape the course of cognitive development during infancy and childhood (e.g., Bandura, 1977, 1986; Vygotsky, 1978; Rogoff, 1990; Farrant and Reese, 2000). Children have a zone of proximal development, that is, the difference between what they are able to accomplish independently and what they can achieve with the help of a more experienced adult (Vygotsky, 1978). High interactional quality between infants and caregivers should provide a scaffold under challenging learning conditions (Wood et al., 1976). High quality parent–child interactions are characterized by parents’ use of appropriate amounts and types of verbal input, emotional responsiveness where parents are sensitive to the developmental needs of the child and the child is engaged, and parents who provide structure and guidance during everyday activities and teaching tasks (DeLoache and DeMendoza, 1987; Rogoff, 1990; Farrant and Reese, 2000; Dodici et al., 2003). The present study examines whether dyadic interactional quality—characterized in this way—is associated with learning from a novel touch screen tool during infancy.

Much of the research in this domain has focused on maternal behavior during parent–child interactions. Mothers’ sensitive and contingent verbal input during dyadic interactions shapes their infant’s immediate phonological patterns (Goldstein and Schwade, 2008) and vocal development over time (Gros-Louis et al., 2014). Other research shows that mothers differ in how they talk about the past with their children, with some mothers being classified as elaborative and others as repetitive (e.g., Reese and Fivush, 1993). More elaborative maternal scaffolding during infancy predicts higher and more diverse productive vocabulary outcomes for infants and preschoolers (Hart and Risley, 1995; Haden et al., 1996; Hoff, 2003; Britto et al., 2006) and increased child engagement and responsiveness to verbal requests (Hudson, 1990). Mothers adjust their verbal scaffold during book reading based on the developmental level of their child (DeLoache and DeMendoza, 1987; Sénéchal et al., 1995). These measures of maternal scaffolding are dependent upon the bidirectional relationship between the parent and child.

As technology created specifically for young children proliferates, researchers have more closely examined parent–child interactions during television viewing and computer storybook reading (Stoneman and Brody, 1982; Lauricella et al., 2009). For example, during a computer book reading task between caregivers and preschoolers, Lauricella et al. (2009) found that when the child operated the mouse, caregivers concentrated on scaffolding the mechanics of the task. Conversely, when the caregivers operated the mouse, caregivers concentrated on scaffolding children’s vocabulary and comprehension of the story.

Variation in interactional quality has also been found in studies of co-viewing during infant-directed programming (Barr et al., 2008; Fender et al., 2010; Fidler et al., 2010). In general, the more parents provided labels and descriptions and asked about the video content, the more likely infants were to vocalize (Fender et al., 2010), to look at the screen (Barr et al., 2008; Fidler et al., 2010) and to interact with the media characters (Barr et al., 2008). In a study on toddler word learning from video, Strouse and Troseth (2014) found that 24-month-olds only transferred a word they learned from watching a video to a real 3D object when a parent provided verbal scaffolding. Taken together, these results suggest that the presence of a contingent, social partner may have important influences on infants’ learning from books, television, and computers.

Back-and-forth responsiveness between infants and their parents shapes infant development. Researchers often use global rating scales to measure the contingent nature of parent–infant interactions. Rather than counting the frequency of behaviors, for global scales researchers make a qualitative rating based on how often a parent or child displays specific behaviors or an interactional style (Brito et al., 2014). Although, responsiveness and maternal structuring have been indexed in a number of different ways, across a wide range of contexts, and with diverse populations (e.g., Bornstein, 1989; Martin, 1989; Barnard and Kelly, 1990; Rogoff et al., 1993; Biringen et al., 1998; Biringen, 2000), the construct consistently predicts cognitive, language, and social outcomes across populations and throughout development (Bornstein, 1989; Tamis-LeMonda et al., 2001; Laible and Song, 2006; Bornstein et al., 2008; Kaplan et al., 2009; Brito et al., 2014).

For an adult’s response to be contingent, it must be sequential to, and dependent on the infant’s behavior. In face-to-face interactions, contingent responses help keep and direct infants’ attention. Joint attention refers to “following the direction of attention of another person to the object of their attention” (Butterworth, 2001, p. 213). Infants’ ability to jointly attend develops gradually across the first 2 years of life. Eye gaze and pointing are simple ways for mothers and infants to respond to or initiate joint attention. By the end of their first year, most infants have learned that interactions are based on reciprocal and interchangeable roles (Shaffer, 1977). However, children’s responses to their mother vary significantly across individual dyads (Martin, 1989; Barnard and Kelly, 1990; Biringen et al., 1998; Biringen, 2000; Easterbrooks et al., 2005).

Contingency is also important for toddlers’ language learning from screen media (Roseberry et al., 2014; Kirkorian et al., 2016). For example, toddlers learned new words from a contingent, video chat interaction as well as from a face-to-face interaction; however, they did not learn the words from a non-contingent, pre-recorded video (Roseberry et al., 2014). Research examining dyadic parent–child interactions has typically focused on parent–infant exchanges during familiar activities such as toy play, feeding time, and book reading. One area that has received less attention is parental *teaching* of infants in novel, supportive contexts.

Interactional quality during maternal teaching, indexed by verbal input, responsiveness, and contingency, is also a major construct that predicts children’s performance on problem solving and puzzle tasks (Maccoby and Martin, 1983; Goldberg et al., 1989; Barnard and Kelly, 1990; Britto et al., 2006; Levine et al., 2012; Fisher et al., 2013). For example, Levine et al. (2012) examined parent–child interactions during puzzle play every 6 months beginning when children were 2 years old. At 4.5 years children completed a mental rotation task. They found that the quality of parent engagement and spatial language use during puzzle play predicted children’s later performance on the mental rotation task.

In general, research examining the role of interactional quality on child learning outcomes has largely relied on older age groups or familiar tasks (e.g., Laosa, 1980; Britto et al., 2006; Fisher et al., 2013); but caregiver teaching has also been examined in infants (Dixon et al., 1984; Brachfeld-Child, 1986; Banerjee and Tamis-LeMonda, 2007). For example, Brachfeld-Child (1986) found that parents use a variety of teaching strategies when asked to teach their 8-month-olds a new skill – putting a cube in a cup – including attention-getting behaviors and pointing, making the test object more accessible and stable, and vocalizing. This research has primarily focused on providing broad descriptions of maternal behavior and child behavior (e.g., persistence) without connecting the teaching to *immediate* infant success on a task (e.g., Britto et al., 2006; Banerjee and Tamis-LeMonda, 2007). Even when immediate success has been measured (e.g., Laosa, 1980), the success rate has been low, suggesting that the task may not have been developmentally appropriate for the age group tested. Finally, both maternal modeling and verbal instruction of the learning outcome are often permitted during the teaching task (e.g., Dixon et al., 1984; Brachfeld-Child, 1986) making it impossible to disentangle children’s ability to complete the task in the presence or absence of explicit modeling.

In order to examine the role of interactional quality on infant learning, a task needs to be devised with two criteria in mind: (1) the infant needs to be able to physically engage in the task and (2) it should be a task in which infants have demonstrated a difficulty in completing on their own. Transfer of learning between 2D and 3D tasks meet these criteria.

Learning to apply knowledge from a touch screen is complex because it involves transfer of learning. Researchers have demonstrated that infants show a “transfer deficit” (Barr, 2010), that is, they have difficulty transferring learning from 2D sources such as books, television, and touch screens to real-world, 3D objects in comparison to learning from live, face-to-face interactions with real objects (e.g., Barr and Hayne, 1999; Anderson and Pempek, 2005; Zack et al., 2009). For example, Zack et al. (2009) used a novel touch screen to examine whether infants would imitate actions modeled on a touch screen device. The experimenter pushed a button either on a touch screen or a real toy to produce an interesting sound (e.g., a honking sound). Using the touch screen device allowed the researchers to examine how flexible infants could be in transferring learning from the touch screen device to the real toy and vice versa. For the 3D/2D condition, an experimenter pushed a button on the 3D toy and infants were given the opportunity to imitate the action on a 2D touch screen image of the toy. Infants saw the reverse for the 2D/3D condition. Infants in baseline only conditions did not view a demonstration before being shown the test 3D toy or 2D touch screen image.

Zack et al. (2009) reported three major findings with their novel touch screen task. First the task has a low baseline for both the 2D touch screen test and 3D object test, a quintessential hallmark for an experimental imitation task (e.g., Barr and Hayne, 2000). Second, infants performed above baseline in all experimental conditions. Finally, although infants performed significantly above baseline, indicating that they could transfer learning between the touch screen and the real toy, they learned significantly less compared to when the demonstration and test both occurred on the touch screen (2D/2D) or on the real toy (3D/3D). In a follow-up study, Zack et al. (2013) found that language cues did not augment infant imitation scores to above original transfer performance on the touch screen transfer task. The touch screen transfer task therefore meets the two criteria: infants could physically engage in the task but the transfer task was sufficiently challenging.

In the present study, we therefore used the touch screen task to explore whether interactional quality predicts infant learning. Mothers were asked to teach the touch screen transfer of learning task to their infants. The touch screen transfer conditions (Zack et al., 2009) were adapted into a semi-naturalistic teaching task. Mothers were given a 3D toy, and a static image of the object presented on a touch screen. Mothers had 5 min to teach their infant that a button on the real toy worked in the same way as a virtual button on the touch screen image (or vice versa). The goal of this study was to examine whether variations in interactional quality between mother and infant predict infants’ ability to transfer learning between 2D and 3D. We predicted that higher interactional quality within the dyad—indexed via verbal input, responsiveness, and structuring—would be associated with greater infant success on the touch screen transfer task.

## Materials and Methods

### Participants

Participants were fifty 15- to 16-month-old (25 males) full-term healthy infants and their mothers. They were recruited through commercially available records, childcare centers, and by word of mouth. Mother–infant dyads were visited in their homes between January, 2008 and December, 2009. Infants ranged in age from 15 months and 1 day to 16 months and 18 days (*M* = 15 months, 16 days, *SD* = 11.0 days). Participants were Caucasian (*n* = 39), Latino (*n* = 3), Asian (*n* = 3), and of mixed race (*n* = 5). The majority of infants were from middle- to upper-class families [rank of socioeconomic status (SEI) using Nakao and Treas (1992) calculation, *M* = 79.7, *SD* = 12.2]. Families were well-educated (parent education *M* = 17.84 years, *SD* = 0.5).

Mother–infant dyads were randomly assigned to one of two conditions: *3D demo object/2D test image* (3D/2D) or *2D demo image/3D test object* (2D/3D). There were 25 mother–infant dyads per condition. The primary language spoken at home and during the task was English for 96% of the sample (*n* = 48). Two mothers spoke in English and Spanish during the teaching task, as this was typical of an interaction in their home. An additional five mother–infant dyads were excluded from the final sample due to equipment failure (*n* = 1), maternal failure to follow study directions (*n* = 2), infant fussiness (*n* = 1) and an inability to transcribe the session (*n* = 1).

### Apparatus

**FIGURE 1:**
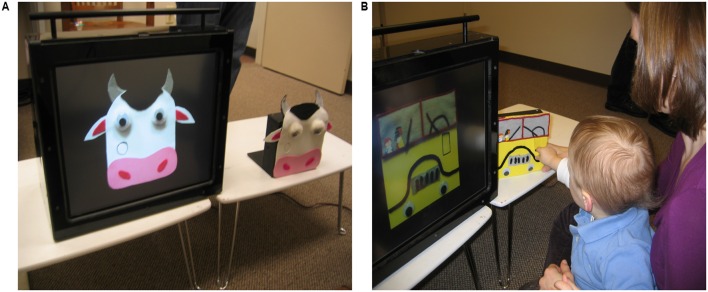
**(A)** 2D touch screen and 3D object experimental stimuli. **(B)** Set-up for the touch screen teaching task.

We created a bus and a cow stimulus from non-commercially available button boxes (Zack et al., 2009, 2013) (**Figure 1**). Mothers were randomly assigned to either the bus or cow stimulus for use in teaching the transfer task.

#### 3D Stimuli

Two button boxes (16.5 wide × 15 tall × 5.5 cm deep) were decorated to create a school bus and a cow. The bus has a slightly recessed rectangle-shaped button (2.2 cm × 3 cm) on the right surface in the middle of the box. Pressing the button produced a horn honking sound. The cow has a slightly recessed circular button (2.2 cm × 2.2 cm in diameter) on the left surface in the middle of the box. Pressing the button produced a cow mooing sound.

#### 2D Stimuli and Touch Screen

Digital photos were taken of the bus and cow 3D button boxes and depicted on a 17 inch LCD touch screen. The button areas were programmed such that pressing the virtual button on the touch screen produced the same sound as pressing the actual button on the 3D toy. The images were equated in size to the 3D object at approximately the same viewing distance.

#### Experimental Set-Up

Two lap tables (each 61 wide × 32 tall × 37.5 cm deep) were placed side-by-side on the floor. The 3D object was placed on one table and the touch screen on the second table (**Figure 1**). Mothers and their infants sat on the floor at the lap tables, facing the 2D touch screen and 3D object. The 3D object and touch screen were covered with a black cloth until the start of the session.

### Procedure

This study was carried out in accordance with the recommendations of the Georgetown University Institutional Review Board with written informed consent from mothers of all subjects. Mothers of all subjects gave written informed consent in accordance with the Declaration of Helsinki. After obtaining informed consent, an experimenter described the study and gave mothers written and verbal instructions. The instructions included a description and illustration of the task set-up, goals, and restrictions. We instructed mothers to teach their infant about the relationship between the 3D object and 2D touch screen image. That is, that a button on the real object works in the same way as a virtual button on a touch screen (or vice versa). For example, a mother assigned to the 2D/3D condition was allowed to interact with or discuss either the 3D object or touch screen. But mothers had one caveat. They could not directly point out the 3D button, push the 3D button, or say push with regard to the 3D object. The mother’s goal was for her infant to figure out the connection between the 2D touch screen and the 3D object. The mother succeeded if her infant pressed the 3D button within the 5-min session.

One experimenter videotaped the session from behind the two lap tables. The mother’s and infant’s face were visible at all times. A second experimenter videotaped the session from behind the mother–infant dyad. The mother’s and infant’s arms and the touch screen and object were visible at all times. The session ended when the infant pressed the button on the 3D object (2D/3D condition) or 2D touch screen image (3D/2D condition), or at 5 min, whichever came first.

### Questionnaires

#### MacArthur Communicative Development Inventory (CDI, Level 1)

Infant short form is an 89-word parent report checklist of words their infant understands and understands and says (Fenson et al., 2000). Percentile rank was determined by the age and gender of the infant for language comprehension and production. Infants’ language ability was within expected norms for 15- to 16-month-olds (*M* = 40.9, *SD* = 32.5).

#### Household and Infant Screen Media Use

Mothers were asked to estimate their daily household screen media use and amount of time their infant was exposed to television on a typical day. Touch screen use was not included in the questionnaire because very few homes had touch screen phones or tablets at the time data was collected.

### Coding – Task Variables

#### Transfer Success

A primary coder scored from videotape whether infants performed the target action (pressing the button) on the test object (2D/3D condition) or test image (3D/2D condition). Transfer score was ‘0’ if the infant did not press the button within 5 min from the start of the session. The transfer score was ‘1’ if the infant did press the button. A secondary coder scored 50% of the sessions; inter-observer reliability was 100%.

#### Latency to Success

Latency to success was calculated from infant’s first touch of the test stimulus to be consistent with previous experimental studies using touch screens (Zack et al., 2009, 2013). Infants who did not successfully transfer on the task received a latency time of 5 min, the maximum amount of time dyads had to complete the task.

### Coding – Maternal Scaffolding

#### Proportion of Diverse Verbal Input

The transcripts were coded to examine how much “new” information the mother provided during the task. An utterance was coded as *diverse* in the transcript if the mother had not provided the same information within the previous 10 utterances. An utterance was defined as *repetitive* if the mother had provided the same content (Reese and Fivush, 1993) within the previous 10 utterances (see **Table 1**). A Pearson product-moment correlation yielded an inter-observer reliability coefficient of 0.96 based on 30% of the sessions.

**Table 1:** **(Left)** A transcript of a diverse interaction. **(Right)** A transcript of a repetitive interaction.

Diverse	Code	Repetitive	Code
What does a cow say?	N	Look at this	N
Moo	N	Look at that	R
And there’s another cow	N	Look at that	R
Look (child’s name)	N	It’s a screen	N
This is how I make him go moo	N	Doesn’t that look like the other toy?	N
And look – 1 cow, 2 cows	N	Doesn’t it look like the other toy?	R
I know it’s so funny	N	It’s yellow	N
Can we make him go moo?	N	Looks like the other toy, doesn’t it?	R

*Each phrase is coded N for new or R for repeated content*.

#### Maternal Modeling

A coder scored each time the mother pushed the button on the demonstration stimulus; the rules of the task stipulated that mothers were not permitted to push the button on the test stimulus. A “button push rate” was calculated to control for differences in session length across dyads. The rate was calculated by taking the total number of times the mother pushed the button on the demonstration stimulus and dividing by the individual session length for each dyad (maximum time = 5 min). Reliability was 89% (κ = 0.76) based on 34% of the data.

#### Maternal Structuring

Maternal structuring was characterized by how often the mother organized her infant’s attention, motivation, and involvement in the task and attempted to teach the transfer task. The dimensions were adapted from other research groups (Goldberg et al., 1989; Barnard and Kelly, 1990; Biringen, 2000). A mother was classified as either providing an *optimal* amount of structure (score = 1) or *too little/too much* (score = 0) structure. Mothers who provided an optimal amount of structure would let their infants be autonomous while also guiding their behavior to reach the goal. For example, 80% of mothers used verbal matching cues to illustrate that a feature on the 2D image was also present in the 3D object. Reliability was 93% (κ = 0.84) based on 30% of the data.

### Coding – Infant Behaviors

#### Infant Button Pushes

A coder scored each time the infant pushed the button on the demonstration stimulus. A “button push rate” was calculated to control for differences in session length across dyads (see Maternal Modeling). The coder also scored when the infant pushed the button on the test stimulus, which was coded as *transfer success*. Reliability was 90% (κ = 0.81) for total number of infant button pushes, based on 20% of the data; however, reliability for transfer success was much higher (100%), based on 50% of the data.

#### Infant Activity Level

Because this study was conducted under semi-naturalistic conditions in infants’ homes, infant activity level varied during the task. Low activity was coded if infants were primarily situated in one location (e.g., on the mother’s lap), whereas moderate activity was coded if an infant frequently moved around the teaching task area. Reliability was 93% (κ = 0.84) based on 30% of the data.

### Coding – Emotional Responsiveness

#### Emotional Responsiveness

To examine the reciprocal relationship between mother and infant, emotional responsiveness was coded on the basis of four global scales: shared focus, turn taking, maternal warmth, and infant involvement (adapted from Laible and Song, 2006; Fidler et al., 2010). For each dimension, dyads were rated on a five-point scale (with 1 = low amount of behavior and 5 = high amount of behavior) and anchor point definitions are provided next. Codes were not assigned for two mother–infant dyads in which the infants successfully transferred in less than 1 min; the session did not last long enough to accurately assess the measures.

#### Shared Focus

High shared focus was defined as a sense of togetherness, shared meaning, and unity with regard to the task; mother and infant “being on the same page.” Low shared focus was defined as the mother and infant being engaged in completely different aspects of the task for the majority of the session, or a child who was engaged in off-topic play for most of the session. Reliability was 81% (κ = 0.74) based on 32% of the data.

#### Turn Taking

High turn taking was defined as the degree to which caregivers and infants engaged in conversational exchanges (verbal or non-verbal back-and-forth) with regard to the task. Low turn taking was defined by the absence of this type of exchange. Reliability was 81% (κ = 0.70) based on 32% of the data.

#### Maternal Warmth

High maternal warmth was defined as a mother’s sensitive, engaging, and affectionate style toward her infant’s affective cues; including promptness and appropriateness of reactions, physical affection, positive affect, tone of voice, and frequent encouragement and praise. Low maternal warmth was defined by frequent instances of frustration with the infant and no instances of encouragement or praise of the infant; a mother going through the motions of the task without engaging the infant. Reliability was 94% (κ = 0.88) based on 32% of the data.

#### Infant Involvement

High infant involvement was defined by consistent infant interactions with the mother and active verbal or non-verbal responses to a mother’s directives or requests. Low infant involvement was defined by an infant being unreceptive to a mother’s directives or requests. Reliability was 94% (κ = 0.91) based on 32% of the data.

#### Total Emotional Responsiveness

An overall emotional responsiveness score was calculated by summing the dyads’ scores for each emotional responsiveness measure (maximum score = 20). Reliability was 88% (κ = 0.82) based on 32% of the data.

## Results

### Analysis Plan

Preliminary analyses indicated that test condition (2D/3D or 3D/2D), average household media use (hours/day) or infant media use (hours/day), infant receptive or productive vocabulary (MCDI), parent education, socioeconomic status, or sex of child (male or female) did not show main effects or enter into any significant interactions. Therefore, these variables will not be discussed further, with the exception of test condition.

### Transfer Success

Infants’ transfer success on the touch screen task was 64% (*n* = 32). Transfer success did not differ by condition; 64% of infants were successful in the 2D/3D condition and 64% were successful in the 3D/2D condition. Although moderately high, transfer performance was well below ceiling.

#### Infant and Maternal Button Pushes

Infants pushed the button more often in the 3D/2D condition (*M* = 2.87, *SD* = 1.75) compared to the 2D/3D condition (*M* = 1.81, *SD* = 1.76). On the other hand, mothers modeled the button push more when the demonstration tool was the novel 2D touch screen image (2D/3D condition, *M* = 3.44, *SD* = 2.22) compared to when it was a 3D object (3D/2D condition *M* = 2.28, *SD* = 1.36), perhaps because the touch screen was a novel tool. That is, mothers adapted their demonstrations to meet the experience level of their infants.

#### Latency to Success

Infants who were not successful on the task (*n* = 18) automatically received the maximum total session time of 5 min. For those who were successful, the average latency to success from the time of first touch of the test stimulus was 1.57 min (*SD* = 1.27 min).

#### Infant Activity Level

Low activity level infants were significantly more likely to successfully transfer (75%; 24/32) than moderate activity level infants (37%; 6/16), χ^2^(1, *N* = 48) = 6.4, *p* = 0.01.

#### Stimulus Type

A chi-square analysis showed that infants tested with the bus (80%) were more likely to succeed than infants tested with the cow (48%), χ^2^(1, *N* = 50) = 5.56, *p* = 0.02.

### Descriptive Statistics

#### Proportion of Diverse Verbal Input

Overall, mothers provided a good verbal teaching context. On average, 62% (*SD* = 12%; range = 39–92%) of mothers’ utterances were new information. This finding is consistent with research examining mothers from middle to high SES, well-educated backgrounds in a teaching situation.

#### Maternal Structuring

Overall, mothers provided either optimal or moderate amounts of structuring in the teaching context. On average, just over half (54.2%) of mothers provided optimal structuring.

#### Emotional Responsiveness

Dyadic emotional responsiveness was on average at least a “3” (0–5 scale) for each individual measure for the infants who did and did not transfer (see **Table 2**). This indicates that high-quality emotional responsiveness within the dyad occurred during approximately half of the session time. On average, total emotional responsiveness within the dyad was 15.56 (*SD* = 3.58).

**Table 2:** Mean emotional responsiveness ratings by infant transfer success.

	Transfer success			
	Infant transfer (*n* = 30)		No infant transfer (*n* = 18)	
Emotional responsiveness	*M*	*SD*	*M*	*SD*
Shared focus	4.27	0.87	3.17	0.99
Turn taking	3.93	0.94	3.11	0.90
Maternal warmth	4.40	0.81	3.83	0.71
Infant involvement	4.37	0.72	3.11	0.90
Overall	16.97	3.01	13.22	3.28

### Interactional Quality

One of the main goals of the study was to examine whether mother–infant dyads exhibited different patterns of interactional quality during a touch screen transfer of learning task. Thus we conducted a K-means cluster analysis technique to classify cases into subgroups based on a set of specific attributes (Easterbrooks et al., 2005): emotional responsiveness, maternal structuring, and diversity of maternal verbal input.

The proportion of diverse maternal verbal input, total emotional responsiveness score, and amount of maternal structuring were chosen to enter into the cluster analysis because prior research has shown positive associations between mothers who respond and adapt to their infants’ behaviors and vary their verbal input to match their infants’ focus of attention, and later brain (Bernier et al., 2016) and cognitive development (e.g., DeLoache and DeMendoza, 1987; Rogoff, 1990; Farrant and Reese, 2000; Flynn and Masur, 2007). The cluster analysis included measures scored for 48 of the mother–infant dyads in the sample using a two-cluster model, as a sample size of 48 is sufficient for classifying cases into two clusters (Stata Manual, 2007). Cluster 1 (*n* = 31), was named *high interactional quality* with maternal teaching characterized as well-structured, a high proportion of diverse maternal verbal input, and high overall levels of emotional responsiveness within the dyad. Cluster 2 (*n* = 17) was named *moderate interactional quality* with maternal teaching characterized as moderately structured, a moderate proportion of diverse maternal verbal input, and moderate levels of emotional responsiveness within the dyad. **Table 3** shows the means for maternal teaching, the proportion of diverse verbal input, and emotional responsiveness as a function of each cluster.

**Table 3:** Maternal structuring, proportion of diverse maternal verbal input and overall emotional responsiveness as a function of interactional quality group.

	Interactional quality group			
	High interactional quality (*n* = 31)		Moderate interactional quality (*n* = 17)	
	*M*	*SD*	*M*	*SD*
Maternal structuring	1.68	0.60	0.18	0.53
Proportion diverse maternal verbal input	0.66	0.09	0.54	0.11
Overall emotional responsiveness	17.77	1.80	11.53	2.21

#### Predictors of Infant Transfer

The second main goal of the task was to examine what specific elements of the task itself or mother–infant behaviors (i.e., interactional quality) may *predict* infant transfer success. Because infants could either succeed on the task or not, logistic regression was used for this analysis. The dependent variable was dichotomous; with ‘1’ indicating infant success on the transfer task and ‘0’ indicating the infant was not successful on the transfer task. The independent variables included were dyads’ classification as high or moderate interactional quality, infant activity level, and stimulus (bus or cow); all variables were dichotomous.

The results of the logistic regression revealed that only the level of interactional quality was a significant predictor of infant success on the transfer task (**Table 4**). Infant activity level and stimulus were not significant predictors of infant transfer success. The significant odds ratio of 20.45 (*p* = 0.01) for interactional quality indicates that infants were 19 times more likely to succeed on the task if they were in a high interactional quality dyad, holding all other variables constant (**Table 4**). The accuracy of the prediction performed by the logistic regression was also evaluated using a classification table. Approximately, 87% of infants who were predicted to be successful on the transfer task were in fact successful. Approximately 72% of infants who were predicted to be unsuccessful were not successful.

**Table 4:** Results from logistic regression analysis of infant transfer success.

	*B*	*SE*	*P*	Odds ratio
Interactional quality group	3.02	1.21	0.01	20.45
Activity level	-0.60	1.25	0.63	0.55
Stimulus	1.23	0.78	0.11	3.42

*B: unstandardized estimates; *SE*: standard error*.

A standard linear regression analysis was also conducted with the same independent variables (interactional quality group, infant activity level, and stimulus) and infant latency to success (from the start of the session) as the continuous, outcome variable. Infants who did not succeed on the task were given a latency of 300 s, the maximum time allowed to complete the task. Initial collinearity diagnostics indicated that all Variance Inflation Factors were ≤2. The overall model for infant latency to success was significant, *F*(4,43) = 6.36, *p* = 0.001, *R* = 0.55, *R^2^* = 0.30. The pattern of results was identical to those found in the logistic regression analysis; only interactional quality group was a significant predictor of infant latency to success. Infants in the high interactional quality group took less time to successfully transfer compared to infants in the moderate interactional quality group.

## Discussion

This study builds on past research examining parent–infant interactions surrounding media use by (1) examining maternal scaffolding measures of verbal and non-verbal behavior, and interactional quality within each dyad and (2) measuring their relation to an immediate infant learning outcome in the context of a novel, touch screen teaching task.

### Interactional Quality and Infant Transfer Success

Interactional quality, as measured by emotional responsiveness, maternal structuring, and diversity of maternal verbal input, significantly predicted infant transfer success. Infants in high interactional quality dyads were more likely to successfully transfer than infants in the moderate interactional quality dyads. In the presence of a supportive social partner, infants were just as successful when mothers were asked to teach from 3D to 2D as they were when mothers taught from 2D to 3D. Interactional quality seems to be especially important for infants because their representational, linguistic, and perceptual systems are still developing; therefore it can be challenging for them to integrate multiple sources of information on their own. This study showed that infants do not easily understand the functional equivalence between a 2D image and 3D object without additional support. In fact, 18 of the infants (36%) failed to transfer between 2D and 3D. This group was marked by lower amounts of emotional responsiveness within the dyad, less maternal structuring, and less diverse maternal verbal information.

#### Diverse Verbal Input

Mothers in high interactional quality dyads provided a higher proportion of diverse information compared to mothers in moderate interactional quality dyads. These mothers would either make a statement (e.g., this is a cow) and immediately elaborate on it (e.g., the cow says moo), or provide new information (e.g., you can push his button). In comparison, mothers in moderate interactional quality dyads did this less frequently, often providing the same piece of information multiple times in a row (e.g., this is a cow, see **Table 1**). Although, all mothers did revert back to providing some of the same verbal information that they used earlier in the task, the mothers of infants who transferred were not as repetitive in the sequencing of their verbal input. It is possible that mothers who varied their verbal input more frequently did so because they were better attuned to their infants’ actions and interest in the task. These findings are consistent with studies examining mothers reminiscing with their preschool-aged children about the past (Fivush and Fromhoff, 1988; Hudson, 1990; Reese and Fivush, 1993).

#### Emotional Responsiveness

High interactional quality dyads were characterized by higher levels of turn taking and synchrony in their interactions. This illustrates the importance of not only the mother, but also the infant’s involvement in the task. It was both the infants’ verbal and non-verbal responses, and the mothers’ sensitivity to their infants’ interests that contributed to the high level of emotional responsiveness. Thus, infants might have benefited more from the verbal and non-verbal input of mothers who timed their behaviors to ensure they had their infants’ attention (Tamis-LeMonda et al., 2001; Flynn and Masur, 2007). Emotional responsiveness consistently predicts future cognitive, language, and social outcomes (Bornstein, 1989; Tamis-LeMonda et al., 2001; Bornstein et al., 2008; Kaplan et al., 2009). Recent research has opened the possibility of a link between the quality of maternal behavior during mother–infant interactions and infant prefrontal brain development, the same area of the brain activated during executive function tasks (Bernier et al., 2016).

Consistent with the present findings, Ayoun (1998) found that the level of maternal responsiveness exhibited by mothers to their 11-month-olds during a free play session was significantly related to how well infants performed on a hidden object and contingency-based touch screen task. Ayoun proposed that infants who have been nurtured in predictable, responsive relationships with their caregivers are more likely to detect relationships between actions and goals in other contexts. Although, Ayoun’s conclusions were speculative, they are consistent with the present findings.

#### Maternal Structuring

High interactional quality dyads were also characterized by mothers who provided optimal levels of structure. These mothers attempted to organize their infants’ attention and interest in the task compared to moderate interactional quality dyads where mothers were more likely to provide too little or too much structure. Mothers’ use of appropriate amounts of guidance and structure during the task is consistent with prior research showing a positive relationship between supportive parent–child interactions and young children’s cognitive development (Rogoff, 1990; Farrant and Reese, 2000; Dodici et al., 2003).

One unpredicted finding was that mothers rarely used verbal matching strategies when teaching. Most mothers provided a verbal matching cue on at least one occasion (e.g., “this cow moos [2D] and this cow moos [3D]),” but half of the mothers did so on fewer than five occasions. Given the correspondence between the 3D object and 2D image, it was surprising that most mothers did not capitalize on the side-by-side presentation of the 2D touch screen image and 3D object to accentuate their similarities. One possibility is that the perceptual similarity between the 2D image and 3D object was an obvious correspondence to the mother and because it was obvious to the mother she may have assumed it was also obvious to the child. Adults seamlessly navigate between 3D objects and 2D media tools (e.g., computers, television, smartphones) in their daily activities so they may be unaware of the difficulties infants face in transfer of learning across dimensions.

### Limitations and Future Directions

There were three overall limitations of the touch screen teaching task that need to be addressed in future studies. First, there were limitations of task complexity and infant age. The one-step action chosen restricted the score to 1 or 0 and is limited to 15- and 16-month-old infants so the findings may not be generalizable to other age groups. Moreover, the types of teaching strategies that mothers employ would be predicted to change with age. Second, although responsiveness and structuring have been assessed in other studies using a 5-min task (e.g., Easterbrooks et al., 2005), it is still a short time period to fully assess these global ratings (Biringen et al., 2005). In this sample, only half of the mothers were still teaching during the fourth minute of the task. The raters also could not be completely blind to infant success on the task because of noticeable variations in session length – many of the infants who succeeded completed the task in less than 5 min time.

A semi-naturalistic task provides a good opportunity to investigate how interactional quality is related to transfer of learning from touch screens. Although, it was important to have an immediate outcome measure in this transfer of learning task, future research should also examine infants’ ability to retain an understanding of the relationship between 2D and 3D by testing infants after a delay. From our data it is unclear how much the side-by-side presentation of the touch screen and 3D object contributed to infant transfer success, although the better transfer performance by infants in the high interactional quality group suggests success was not simply related to the nature of the task set-up. In future studies, specific aspects of the task that might have improved transfer can be experimentally manipulated to test their effects. Future transfer of learning studies should examine the facilitative effects of a side-by-side presentation of the 3D object and 2D touch screen image, increase the length of the demonstration and/or test, manipulate the amount and type of verbal and non-verbal input (e.g., pointing), and control the level of responsiveness provided by the mother or experimenter. Future studies should also explore whether infant success on the touch screen task is related to infant success on other 2D–3D transfer of learning tasks, such as learning from books or television.

### Implications

Media use surveys show that infant touch screen use is on the rise. For children in the United States under the age of 2, 38% have used a mobile device; 51% of children have used smartphones and 44% tablets at least once by 2 years of age (Rideout, 2013). In a questionnaire study with low-income, minority families, Kabali et al. (2015) found that of children currently under age one, 92% had used a mobile device (e.g., smartphone, iPad, or tablet) whereas only 40% of current 4-year-olds used a mobile device before 1 year of age. This difference reflects an increase in mobile device use over 4 years time. They also found nearly 77% of children used mobile devices daily by age 2. Young children’s widespread use of touch screens also extends beyond the USA (Neumann, 2014; Cristia and Seidl, 2015; Ahearne et al., 2016). Taken together, these findings show that in some communities, families are using interactive media early and often.

The benefits of high quality interactions during everyday activities such as feeding and book reading are consistently related to children’s later cognitive and social development (e.g., Rogoff, 1990; Farrant and Reese, 2000; Hoff, 2003; Bernier et al., 2016). This is important with regard to infant learning from 2D media sources. If supportive interactions with infants during daily activities foster positive growth and development then it is reasonable to expect high interactional quality to be necessary for infant learning in media contexts with more novel forms of technology.

Hirsh-Pasek et al. (2015) proposed that we should draw from the Science of Learning field to understand how we can best promote children’s playful learning from interactive devices. There are four components that need to occur for apps to be educational: cognitively active, engaged, meaningful, and socially interactive. These recommendations for app development arise from their general principles of guided play (for review see Weisberg et al., 2016). When infants successfully transferred, mothers were more likely to be cognitively active in that they promoted purposeful interaction; they kept their infant on task; they scaffolded their infant’s existing knowledge; and they served as a contingent partner.

In sum, transfer of learning between 2D images and 3D objects is challenging for young children. The present findings suggest that for families in the digital age, the context in which infants learn from interactive technology is pivotal for transfer of learning between 2D touch screen and 3D sources. This research suggests that infants require input from an engaged, responsive social partner if they are going to understand the functional relationship between 2D and 3D sources. Parents should be educated about the challenges infants face in transferring information between touch screens and objects in their physical world. They should be encouraged to co-use media rather than rely on the touch screen as a stand-alone educational device, and use effective scaffolding techniques to enhance infants’ transfer of learning from touch screens. Media has the potential to serve as an effective teaching tool that enhances learning in young children when used in supportive parent–child contexts.

## Author Contributions

This data was originally published as part of EZ’s doctoral dissertation, *Infant transfer of learning across 2D/3D dimensions: a touch screen paradigm*. EZ developed the study concept, designed the study and data analysis plan, collected, analyzed and interpreted the data, and drafted the manuscript. RB developed and designed the study concept and data analysis plan, interpreted the data, and provided critical revisions. Both authors approved the final version of the manuscript for submission.

## Conflict of Interest Statement

The authors declare that the research was conducted in the absence of any commercial or financial relationships that could be construed as a potential conflict of interest.
